# Could Prolonged Usage of GPS Navigation Implemented in Augmented Reality Smart Glasses Affect Hippocampal Functional Connectivity?

**DOI:** 10.1155/2018/2716134

**Published:** 2018-06-13

**Authors:** Iveta Fajnerová, David Greguš, Jaroslav Hlinka, Tereza Nekovářová, Antonín Škoch, Tomáš Zítka, Jan Romportl, Eva Žáčková, Jiří Horáček

**Affiliations:** ^1^National Institute of Mental Health, Topolová 748, 250 67 Klecany, Czech Republic; ^2^Institute of Computer Science, Czech Academy of Sciences, Pod Vodárenskou Věží 271/2, 182 07 Praha 8, Czech Republic; ^3^Man-Machine Interaction Dpt., New Technologies-Research Centre, University of West Bohemia, Univerzitní 8, 306 14 Plzeň, Czech Republic

## Abstract

**Background:**

Augmented reality (AR) glasses with GPS navigation represent the rapidly evolving technology which spares (and externalizes) navigational capacities. Regarding the expected everyday usage of this device, its impact on neuroplastic brain changes and navigation abilities should be evaluated.

**Aims:**

This study aimed to assess possible changes in functional connectivity (FC) of hippocampus and other brain regions involved in spatial navigation.

**Methods:**

Thirty-three healthy participants completed two resting state functional magnetic resonance imaging (rsfMRI) measurements at the baseline and after 3 months. For this period, the experimental group (n = 17) has had used AR device (Vuzix M100) with incorporated GPS guidance system during navigation in real world. Participants from the control group (n = 16) have not used any GPS device while navigating during walking. The rsfMRI FC of right and left hippocampi was analyzed using a seed-driven approach. Virtual city task was used to test navigational abilities both before and after the usage of AR device.

**Results:**

We identified strong functional coupling of right and left hippocampi at the baseline (p < 0.05, FDR corrected). Mild changes in bilateral hippocampal FC (p < 0.05, FDR uncorrected) were observed in both assessed groups mainly between the bilateral hippocampi and between each hippocampus and temporal regions and cerebellum. However, the experimental group showed FC decrease after three months of using GPS navigation implemented in AR glasses in contrast to FC increase in the control group without such intervention. Importantly, no effect of intervention on navigational abilities was observed.

**Discussion:**

Our observation supports the assumption that externalization of spatial navigation to technological device (GPS in AR glasses) can decrease the functional coupling between hippocampus and associated brain regions. Considering some limitations of the present study, further studies should elucidate the mechanism of the observed changes and their impact on cognitive abilities.

## 1. Introduction

The extremely rapid advances in new technologies may lead to unprecedented modifications of both human bodies and cognitive capabilities. The field of Human Cognitive Enhancement (HCE), addressing various interventions into human cognitive capabilities, potentially affects our everyday life [1]. However, these emerging HCE technologies may transform our cognitive faculties and behavioral patterns in both beneficial and harmful way. Contrary to the formalized procedures and regulation in clinical pharmacological research and testing, the evaluation processes ensuring safety of novel HCE devices are not standardized and psychological and medical risks of these technologies may occur [2].

The augmented reality (AR) glasses (e.g., Vuzix, Google glass) represent the prototypical example of rapidly evolving technology which affects how we perceive the reality we live in. The screen of AR glasses serves as an interface between physical and digital domains enabling experience of the overlap between the real and virtual environment on a daily basis. Since these wearable devices will be most likely widely available within the next few years, it is important to objectively track the short-term as well as long-term health consequences of using them. Regarding their expected everyday usage and the facts that this device could both spare some cognitive capacities (e.g., memory) and produce new demands (e.g., frequent eyes accommodation), the impact of AR glasses on various neurophysiological parameters should be intensively evaluated. Even when Gamberini et al. found mainly positive user experiences with AR glasses, authors express concerns about possible issues related to visual fatigue [3]. On the other hand, a recent study [4] found that AR wearable displays have the potential to reduce mental workload required for navigation when compared to hand-held devices (smartphones). Yet, to our knowledge, studies on physiological and psychological consequences of long-term AR glasses are completely lacking.

This study addresses primarily the possible neurobiological effects of AR devices in regard to “externalization” of our capabilities into the wearable device. The expected popularity of AR glasses is in line with the growing usage of other GPS devices. GPS technology enables us to easily navigate in new environments; however it may in fact negatively affect spatial knowledge obtained during such guided navigation [5] by effectively disengaging our attention from the navigated environment [6] and thus ‘turning off' some areas specific for navigation [7–9]. Thus, we can speculate whether wearable GPS devices used on daily basis may lead to some general decline of spatial abilities (and their neuronal substrate).

The influence of long-term daily navigation (without GPS) on human brain was firstly studied in London taxi drivers. These pivotal studies showed that regular navigation demands in complex environments may lead to increase of the volume of hippocampus (HPC) which is involved in spatial memory [10–13]. These findings document the neuroplastic changes in areas involved in learning and memory. It has been speculated that using spatial memory while navigating regularly not only may improve the function of the HPC but also could help to slow down cognitive impairment as we age, while long-term reliance on GPS may in contrary reduce HPC function [7, 8]. In agreement with this idea, we speculate that the long-term usage of GPS devices can have the opposite effect on brain structure and function, as human cognitive abilities used during navigation are externalized to some GPS device and are not used on everyday basis. Similarly, prolonged usage (for weeks/months) of GPS device could elicit some changes in brain activity and connectivity of the involved brain areas [14]. To our knowledge, no research has so far addressed this issue.

This study aimed to assess possible changes in the resting state functional magnetic resonance imaging (rsfMRI) functional connectivity (FC) of brain regions involved in spatial navigation. Primarily, we hypothesized that the three-month navigation using GPS in AR glasses decreases FC of the right hippocampus (HPC), which is specifically associated with visuospatial memory [15, 16], with other areas involved in navigation process, such as parahippocampal gyrus (especially its posterior part), retrosplenial cortex, lateral and medial prefrontal cortex, inferior part of the parietal lobe, striatum, cerebellum, and midline structure of precuneus [17–22].

The FC of the left HPC (tested to ensure specificity of possible FC changes observed in the right HPC) should not be affected, as it is not specifically involved in spatial memory and was reported mainly in retention of verbal material [15, 23] or in episodic memory [16]. In respect to cognitive effects, we hypothesized that the usage of GPS device during everyday spatial navigation will negatively affect spatial abilities in GPS-trained participants from the experimental group along with the growing dependency on the device.

## 2. Methods

### 2.1. Subjects and Procedure

Healthy right-handed participants (n = 44) have been recruited for the study using an online questionnaire aimed at HCE technology. All participants expressed interest in new technologies and participation in the study. They were assigned to experimental or control group based on their readiness to wear and intensively use the smart glasses for the period of three months (e.g., participants with glasses have been assigned for the control group). From the original set of volunteers, six participants did not finish the study and five volunteers have been excluded from the analyses due to movement artifacts or low quality of rsfMRI data from one of the sessions. Thirty-three participants (27 males and 6 females, Mean_age_(SD) = 28.8 (7.1), education stage: 3.5 (1.0) ranging from tertiary education (3) to master level (5)) finished two repeated assessment sessions, first prior to the intervention (baseline, TEST session) and after 10-12 weeks of intervention (RETEST session). The experimental group (n = 17; 14 males and 3 females; M_age_(SD) = 28.8 (8.1); M_edu_(SD) = 3.4 (0.9)) was instructed to wear AR device Vuzix M100 Smart Glasses (Vuzix Corporation, NY, USA). Participants were instructed to use actively the incorporated GPS guidance system (Osmand application) during everyday navigation in real world (in unknown locations if possible) for the whole period of 3 months at the minimum of 3 hours per week. The maximal usage was not assigned. The participants from the control group (n = 16; 13 males and 3 females; M_age_(SD) = 28.7 (6.2); M_age_(SD) = 3.6 (1.1)) have been also encouraged to navigate in new places as often as possible; however they did not use any HCE device while navigating. Before the study, all participants were ophthalmologically examined to exclude sight defects interfering with their ability to use wearable GPS device. Informed consent was obtained from all participants and the study was approved by the Ethical Committee of the National Institute of Mental Health (NIMH). All subjects were financially rewarded for their participation in the study.

### 2.2. Classification of the Navigational Activity from Incorporated GPS Application

To control the real time of actively used GPS navigation incorporated in AR glasses, we classified the activity of each user based on logs created by the navigation app generated from device sensors: accelerometer, gyroscope, magnetometer, and virtual sensor compass (in approximately 70 ms interval) and GPS (in 15 s interval). Participants from the experimental group (n = 17) wear the smart glasses device, M_logTime_ = 52.5 hours (range between 27 and 111 hours), and the GPS application was used, M_GPStime_(SD) = 33 (7.2) hours. From these data we attempted to identify following events:* walking (*periodic movement of the device with frequency of 1 to 4 Hz) and* wearing head mounted device versus carrying it in a pocket or bag. *(To determine whether participant wore the device on head we assumed that head mounted device is almost elevated with the Earth surface and so the* z*-axis is nearly perpendicular to it. In terms of pitch and roll of the device this means they are both zero for both left and right eye as the software of the device automatically flips the sensor readings according to the device position. We calculated pitch and roll of the device from accelerometer and gyroscope readings and cut both series into approximately 4 s frames. We assumed participant wore head mounted device when their maximums within the frame were less than 45°.) Similarly, four different movement speed cases have been identified from GPS records:* stationary* (speed was under 1.5 km/h),* moving at walking speed* (speed was above 1.5 and below 10 km/h),* riding a bicycl*e (speed was between 5 km/h and 30 km/h), and* driving in a car *(speed was above 30 km/h). The threshold method was used for classification of these events and movement speeds. We used a combination of these events to classify user's activity [24]. For example, users were considered riding a bike when they were moving at “bicycle speed”, no* walking event *was detected, and they were wearing device on head. As proper usage of AR glasses required by the study we only considered events when the participant* was wearing head mounted device,* was receiving position updates from GPS, and was walking or riding a bicycle (n=17; M_GPSwalk_(SD) = 14.9 (8.8) h).

### 2.3. Behavioral Assessment of Navigational Abilities

Spatial navigation skills of all participants have been evaluated in the TEST and RETEST sessions using the following procedure.

Effects of AR glasses wearing on navigation performance were tested in the virtual city task (VCT) [25]. The virtual environment (VE) of a small city was developed using the Unity game engine software [26]. Individual tasks of the VCT required the participants to navigate using a GPS-like schematic map of the environment in two conditions, either with marked trajectory (route following) or without it (wayfinding). In total participants visited 42 virtual city locations in pairs (such as hospital and university, etc.). Navigation in the virtual city was subsequently tested in a recall session with no GPS map present. All pairs of recalled locations differed from the pairs used during the pretraining. Maximal duration of one trial was set to 60s. Behavioral performance in VCT was evaluated in terms of pointing error (angular difference between the estimated and correct direction) and path efficiency (ratio of minimal and real trajectory). Finally, all participants have been asked to write down all remembered locations into a blind schematic map of the environment. For more details on methods see [25].

### 2.4. The rsfMRI Data Acquisition

Data were acquired on 3T Siemens Prisma MRI scanner (Siemens, Erlangen, Germany) equipped with a standard head coil. The rsfMRI was measured with a gradient echo-planar sequence (GRE-EPI, TR=2000 ms, TE=30 ms, flip angle 70°, bandwidth 2 170 Hz/pixel, iPAT 2, FOV=1344mm×1344mm, matrix size 64x64, voxel size 3x3x3 mm, each volume with 37 axial slices with an interslice gap 3, a total of 300 volumes). Whole brain anatomical scans were also acquired using a 3D T1-weighted magnetization-prepared gradient echo sequence (MP-RAGE), consisting of 240 sagittal slices with resolution of 0.7 x 0.7 x 0.7 mm3 (TR/TE/TI=2400/2.34/1000 ms, FOV=224 mm), which was used for spatial normalization and anatomical reference.

### 2.5. Preprocessing, FC Analysis, and Statistics

Functional connectivity was analyzed using a seed-driven approach with CONN version 15.h connectivity software (www.nitrc.org/projects/conn/). The fMRI data were corrected for head movement, registered to MNI standard stereotactic space by a 12-parameter affine transform maximizing normalized correlation with T1-weighted images, spatially smoothed with a Gaussian kernel (8 mm at full width half-maximum). Physiologic and other spurious sources of noise (signal from a region in the cerebrospinal fluid, white matter, and the whole brain signal) were estimated using the implemented component-based method and removed together with movement-related covariates [27]. The residual BOLD time series were band-pass filtered over a low-frequency window of interest (0.008–0.09 Hz).

The functional connectivity analysis proceeded in two steps to establish first the overall hippocampal functional connectivity and then to study its changes due to the experimental manipulation. In both steps, we used the combined Harvard Oxford Atlas (106 cortical and subcortical ROIs) and Automated Anatomical Labeling (26 cerebellar ROIs) provided with the CONN toolbox. The connectivity of the right hippocampal region to the remaining atlas regions (ROI-to-ROI) was estimated by computing Pearson correlation coefficients between the residual BOLD time courses and further converted to approximately normally distributed Z scores using Fisher transformation. Pearson correlation was previously shown to be a sufficient measure of functional connectivity for ROI-to-ROI fMRI data analysis [28]. Using ROI-to-ROI approach is more robust as it alleviates the multiple testing problem typical for seed-to-voxel FC analysis. The baseline FC connectivity for each region pair was tested by a one-sample t-test applied to the pooled population of both groups (in the baseline condition before experimental intervention). The intervention effect was tested using a random-effects full factorial model, particularly testing the interaction between the groups (experimental group with AR glasses intervention and control group) and time (baseline TEST session 1 versus RETEST session 2 after intervention). In order to improve interpretability of the observed interaction effects, the simple intervention effects within tested groups were tested by t-test for dependent samples which was used for each of the tested groups. To assess the effect sizes for individual analyses Cohen's d was applied. The same analysis was repeated for the left hippocampal region.

For the initial HPC FC mapping we considered as significant only findings at FDR (False Discovery Rate) corrected p-level 0.05. The subsequent analysis of the intervention was limited only to functional connections confirmed in the previous step. However, this still involves a multitude of functional connections, in which the change should be tested. Given the relatively short period of wearing the device and only small sample size in this pilot study, we expect that the effect size may not be large enough to provide sufficient power for detection of the effect if conservative correction for multiple testing (across a family of tested functional connections) is applied. We therefore frame the analysis as only exploratory and report all interventions effects reaching the uncorrected p ≤ 0.05 level. Note that this leads to testing large number of hypotheses (albeit decreased by the first analysis step that limits the testing only to established functional connections), among which we expect on average five percent false positive test results. With this on mind, the results of this analysis should be considered exploratory and interpreted with caution and require validation by targeted tests in future studies.

Statistica software v9.1 was applied in behavioral data analysis; significance level was set to p < 0.05. To test between-group differences in age and education level Student's t-test was applied; Cramer's V test was used to test between-group differences in sex distribution. The 2-way ANOVA with repeated measures (group x session) was used to analyze the effect of intervention (AR glasses usage) on navigational abilities. Spearman rank order correlations have been calculated for the association between the GPS/AR glasses wearing time in active mode and FC change (calculated as a difference between TEST and RETEST z-Fisher-transformed connectivity coefficients) of the right HPC in the experimental (AR glasses) group.

## 3. Results

### 3.1. Demographics and Behavioral Data

The experimental group did not differ from the control group in any of the demographic variables (age: t(31) = -0.06, p = 0.95; Education level: t(31) = 0.53, p = 0.60; sex: Cramer's V = 0.01, p = 0.94).

The moderate usage of the AR glasses (around 3 hours per week) during navigation in real environment for 3-month long period did not significantly affect navigational abilities of the tested participants. We found no significant interaction between group and session variable (2-way ANOVA) in any of the assessed parameters (p > 0.05) in virtual city task (path efficiency: pretraining (F(1,28) = 1.71, p = 0.20) and VCT recall (F(1,28) = 0.98, p = 0.32). The experimental group showed improved ability of direction estimation (towards start position) in virtual experiments. Nevertheless, this effect was found only in case that GPS map was present during navigation trial (pointing error during pretraining in VCT (F(1,28) = 5.93, p = 0.02). Such effect was not observed (p > 0.05) in case the GPS-like map was not available during navigation (pointing error in VCT recall (F(1,28) = 1.02, p = 0.32)).

### 3.2. Resting State fMRI

#### 3.2.1. Baseline Hippocampal FC

Before the intervention (baseline, TEST session 1) the right HPC showed significant (p < 0.05, FDR corrected) functional coupling with the left HPC and bilaterally with several areas of temporal lobe (fusiform and parahippocampal gyrus), amygdala, cerebellum, precuneus, and thalamus. In addition, some moderate positive correlations have been observed with areas of parietal and frontal lobes and some negative correlations have been found with parietal areas of supramarginal gyrus. First ten strongest functional associations out of 63 significant ROIs for right hippocampus (60 for left hippocampus, respectively) are reported in Table 1 (for complete results see Supplementary Table 1).

#### 3.2.2. Intervention-Related Changes in Hippocampal FC

**Figure 1:**
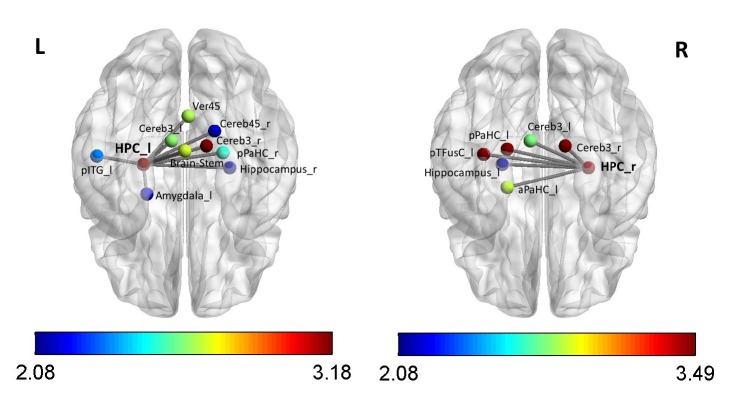
Decrease of right (R) and left (L) HPC functional connectivity (between the RETEST and TEST session) in the experimental (GPS/AR glasses) group (compared to control group) in the inferior view (p<0.05, uncorr.). The color bar represents the statistical power expressed in absolute T values. For anatomical labeling abbreviations see footnote of Table 2.

The subsequent analysis revealed intervention-related FC changes (interaction between group and session) in both hippocampi. In particular, out of the 63 (60) tests for intervention effects on the FC of the right (left) hippocampus, we identified 6 (10) changes in the HPC connectivity, mostly with several cortical structures mainly in temporal lobe and in cerebellum (p<0.05, uncorrected; for details see Table 2 and Figure 1). This is higher number than the expected false positive rate of five percent (that should lead to about three false positive detection results on average for each seed), albeit due to statistical dependence between the hypotheses explicit statistical evidence of this is not available.

**Figure 2:**
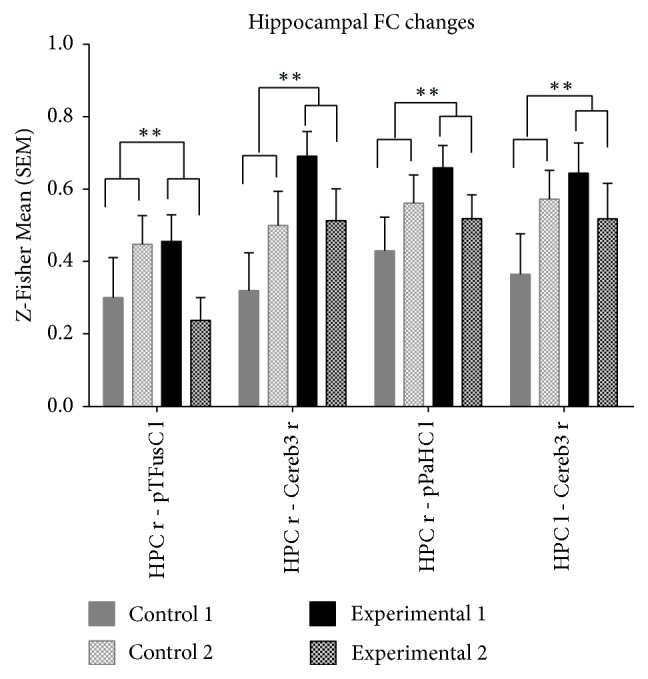
The average z-Fisher-transformed connectivity coefficients between the right/left hippocampus and ROIs (four strongest FC changes (p<0.01) have been selected according to 2nd-level group*∗*session interaction reported in Table 2) displayed separately for each group and session (mean, SEM).* HPC r/l (hippocampus, right/left); pTFusC_l (posterior part of the fusiform cortex, left); Cereb3_r/l (cerebellum lobe III, right/left); pPaHC_l (posterior parahippocampal cortex, left); R: right; L: left; (significant group versus session interactions: ∗∗p<0.01, ∗p<0.05).*

Importantly, the observed changes consist of a mild but significant decrease of the right HPC functional coupling in the experimental group after GPS/AR glasses intervention (see Supplementary Table 2) and mild increase of FC in the control group (see Supplementary Table 3). For visualization purposes only the four strongest effects (p < 0.01) are presented in Figure 2. Notably, two of the areas with intervention-related connectivity change have been in top 10 ROIs showing strongest FC with right hippocampus (posterior parahippocampal cortex and left HPC). Interestingly, a similar effect of intervention has been found also for connections originating from the left HPC.

Moreover, to analyze possible relationship between observed FC changes and intervention with AR glasses, we tested the correlation coefficients between intervention duration and the four strongest FC changes identified (see Figure 2) and additionally the change of functional coupling between both hippocampi. We identified significant Spearman correlation coefficient between the duration of active usage of the AR glasses in head mounted mode and FC changes of the right HPC with the posterior portion of the left parahippocampal cortex (r_HPC_r/pPaHC_l_ = 0.502, p = 0.040). (*It is worth mentioning that the strongest correlation has been found in the region of posterior portion of PaHC known to be involved in visuospatial processes and thus hypothesized to be affected by the GPS usage*). Despite moderate correlation coefficient no significant association was identified for the FC change observed between the right and the left HPC (r_HPC_r/HPC_l_ = 0.453, p = 0.068). Importantly, we did not identify any significant correlation (p > 0.05) between duration of active usage of the GPS in head mounted mode and FC changes of the right HPC with other selected regions of the posterior part of the left fusiform cortex (r_HPC_r/pTFusC_l_ = 0.284) and right cerebellum (r_HPC_r/Cereb3_r_ = 0.336). Similarly, no significant correlation was observed for the FC change between the left HPC and right cerebellum (l_HPC_r/Cereb3_r_ = 0.162).

## 4. Discussion

The main finding of this study, decrease in functional coupling of right hippocampus after 3 months of using GPS navigation implemented in augmented reality smart glasses, is in line with our hypothesis. On the other hand, contrary to our original hypothesis, the FC of left hippocampus has been affected as well. However, in the absence of previous research on functional connectivity changes of hippocampus due to use of wearable devices, we have tested all established functional connections of the studied hippocampal regions, amounting to 63 functional connections of the right hippocampus and 60 tests for the left hippocampus. The analysis showed decreases in functional connectivity of the right (left) hippocampus towards 6 (9) other brain regions at the p<0.05 uncorrected level. Of course, given the amount of connections to be tested, none of the associations was strong enough to constitute a statistically significant result after application of a conservative multiple testing procedure. Nevertheless, the validation of the highlighted FC changes towards identified brain areas could represent suitable target of future studies.

These preliminary observations support the assumption that externalization of some mental capacity (spatial navigation) to technological device (GPS in AR glasses) has measurable neurobiological consequences. Our findings mirror (in a opposite way) the hippocampal volume increase reported in taxi drivers [10–13]. Such remodelations impacting regional brain volumes or connectivity are likely mediated by neuroplasticity as documented by changes in cortical representation in adult brain [29, 30]. The adult neuroplasticity was demonstrated in both nonhuman primates and humans in response to environmental demands and a wide range of cognitive requirements [31–33] such as spatial representation skills [11, 12]. Experience-induced structural and functional changes in plasticity may occur at any part of the ontogeny [34, 35] and after relatively short exposure ranging from 10 to 12 weeks [36]. They are implemented through mechanisms such as axonal remodeling, growth of new dendritic spines, synapse turnover, and hippocampal adult neurogenesis. Such modification may also affect adult brain connectivity [30].

The HPC functional connections to numerous cortical and subcortical areas documented during the baseline condition (before intervention) are in agreement with previously reported dense hippocampal neuronal network and its central role in cognition [37]. Moreover, many of these areas, namely, parahippocampal cortex (posterior part), posterior cingulate cortex, medial prefrontal cortex, anterior cingulate cortex, inferior parietal lobe, precuneus, thalamus, striatum, and cerebellum, are directly involved in spatial learning, memory, and navigation processes [16, 21].

Contrary to our original hypotheses on more pronounced effect on the right than on the left HPC, the GPS/AR glasses usage in experimental group decreased FC of both hippocampi in a similar extent. This finding may be related to the following reasons. First, left hippocampus shows specialization for both episodic [16] and verbal memory [15, 23] and these domains are marginally involved in our AR glasses protocol. For example, verbal material is directly administered by GPS device, while displaying names of streets and locations or auditory directional information. Second, both hippocampi are partially interconnected through dorsal hippocampal commissure (although this connection is limited) [38] and thus functional changes in one HPC might affect the contralateral part as well [39]. Strong interhippocampal FC is also documented by our data. Nevertheless, the reported simple effects (see Supplementary Tables 2 and 3) may signal some lateralization that could be subject of further research.

In respect to specific role of the right HPC in spatial memory and navigation, apart from changes observed in FC between the right hippocampus and the left posterior parahippocampal cortex (pPaHC), no changes have been observed in hippocampal FC with other brain areas related to navigation, such as retrosplenial cortex, parietal lobe regions, or precuneus. We can only speculate that prolonged (three months) GPS usage was not sufficient to elicit changes in those areas which have lower or indirect FC with HPC. Given the intermediate level of compliance of the subjects in this study, the future studies should carefully control the real duration of the GPS use in the active head mounted mode.

Of particular interest is the unexpected mild increase of right HPC FC in the control group. This effect could be produced by the active spatial navigation as control subjects were also instructed to navigate in new places as often as possible but without any GPS device. However, such effect cannot be confirmed given the missing navigation records for the control subjects. Nevertheless, the unbalanced baseline hippocampal FC in both tested groups (see Figure 2) suggests other factors that should be considered. Despite rigorous recruitment of the participants with comparable age, education, and interest in new technologies including GPS devices, their readiness to wear and intensively use the smart glasses for the prolonged time period might affect group assignment and thus the FC results obtained at the baseline. While group differences in FC baseline are accounted for by the statistical design and we only interpret interactions between group and session, future studies should attempt to address this possible confounding factor.

Interestingly, intervention-related changes have been identified mainly in the interhemispheric connections (Figure 1), including functional coupling of the two hippocampi. This finding is congruent with previous studies, which reported that interhemispheric hippocampal FC during rsfMRI predicts individual differences in memory performance [40, 41]. The contralateral hippocampal formation coupling was demonstrated in rsfMRI [42, 43]. The observed decrease of left and right hippocampal functional coupling in our study could thus be related to the externalization of spatial abilities to the GPS/AR glasses device. This assumption is in agreement with the fact that mainly posterior divisions of the fusiform and parahippocampal gyri have been affected. These areas are known to be involved in spatial contextual memory [44, 45] and thus might be specifically spared by GPS navigation. We can only speculate about the GPS/AR glasses induced decrease of right/left HPC, cerebellum coupling affected mainly for regions (Cereb3) within cerebellar Lobe III. This finding could be mediated by active and repetitive leg movements [46] during walking with wearable GPS device.

The observed alterations in hippocampal FC were not connected with substantial behavioral effect as the participants from the experimental group did not show any decline in ability to effectively navigate after the intervention with AR glasses when tested in virtual city environment. Congruently, it was documented previously that physiological changes in functional connectivity may precede cognitive and long-term structural changes due to present compensatory mechanisms [47]. Nevertheless, the lack of direct (negative) impact of decreased right HPC FC on navigation abilities might be due to short duration of the applied intervention and/or limited usage of AR glasses by the participants.

Further studies are needed to investigate the underlying mechanism of the observed FC changes. Specifically, the magnetic resonance spectroscopy (MRS) could elucidate whether natural navigation sparing changes in HPC FC are connected with alterations of neuroplasticity-related hippocampal glutamate-glutamine levels as documented for cognitive performance [37].

### 4.1. Limitations

There are some limitations to the present study that should be pointed out.* First*, the participants in the experimental group did not use the GPS navigation implemented in AR glasses as intensively as it was desired by the protocol. They reported as a reason almost exclusively the technical problems with the AR glasses. Due to technical issues with operating system the Vuzix M100 glasses have been replaced in five subjects during the intervention. Some other issues have been reported using user experience questionnaires (six subjects reported also inaccuracy of GPS signal or sudden crash of the GPS guide—Osmand application—during active outdoor navigation, two subjects complained about an unpleasant noise generated by the device, and seven subjects reported uncomfortable or insufficient placement of the device on the head during movement); for more details see report in [24]. We assume that increased wearing time of the GPS/AR glasses could bring more pronounced connectivity changes and possibly also behavioral effects in tested spatial abilities. Our results could be strengthened by the association between the degree of change in hippocampal connectivity and intensity of AR glasses usage. Even with the above reported significant correlation between specific FC changes of the right HPC and posterior parahippocampal cortex and the duration of AR glasses usage in active mode, other strongest FC changes did not reach significant correlation. Therefore, we cannot argue that the observed effect on FC is exclusively associated with the GPS device usage and observed changes in FC should be therefore interpreted cautiously. However, the observation of moderate correlation between GPS usage and FC of right HPC with the posterior portion of the parahippocampal cortex involved in spatial navigation processes supports our primary hypothesis.

*Second*, we have used a region-to-region functional connectivity analysis approach. Applying a widely used AAL and/or Harvard Oxford atlas, it provides a good signal-to-noise ratio and relatively robust reproducibility of the results compared to a seed-based voxelwise connectivity analysis on the whole brain [28]. On the other side, anatomical atlases (such as AAL) suffer from suboptimal sensitivity due to averaging signal across functionally inhomogeneous areas; this is a similar trade-off that related to large spatial smoothing. Replicating the analysis in a more detailed, possibly functionally informed atlas or with a seed-based or ICA-based approach to FC is a potential avenue of future research.

*Third*, the intervention effects on hippocampal FC were not strong enough to survive conservative correction for multiple comparisons. (*Note: concerning the statistical testing of the intervention effect, we have some albeit not fully conclusive evidence that it is not likely attributable solely to false positives. Overall, we have observed 15 significant results out of 122 hypotheses tested (total numbers decreased by one because one of the connections is between the right and left hippocampi). The probability of observing 15 or more false positives instead of the expected 6 (=~122∗0.05) under independence of the tests is p=~0.0012 (derived from binomial distribution). This strongly suggests that these do not all constitute false positives. However, the tests are not necessary fully independent, and thus such comparison to binomial distribution is not an exact statistical test. A good alternative is nevertheless not readily available, apart from computationally very demanding permutation procedures.*) Clearly, the statistical power in the current study suffers from small sample size. This is natural in a situation of a pilot study aimed at exploring the effect of a logistically costly intervention. Moreover, while we hypothesized that the connectivity of hippocampus is most likely to be affected, the current literature does not provide a strong support concerning which of the hippocampal connections might be altered by the intervention. We therefore chose exploring a multitude of connections and reporting the uncorrected results as a suggestion for the most likely affected connections that should be targeted in future studies. The alternative strategies would include the following: stronger intervention (leading to problem with recruitment and adherence to experimental procedures), larger sample size (unfeasible given the effective budget allocations for a pilot study in an unchartered research territory), preselecting target connections (possible, although, given the lack of convergent prior knowledge in this field, we may also miss important effects), and applying some advanced methods for dealing with the multiple testing problem in a situation of many dependent variables (i.e., the dimension of the data could be reduced before the testing by some clustering procedure or principal component analysis, alleviating the multiple testing problem; such options are however not implemented in standard commonly used neuroimaging data analysis packages and would lead to further technical issues that would render the analysis methodologically too sophisticated and difficult to interpret, reproduce, and transfer to common neuroscientific context).

Despite the above-mentioned limitations, the observed results are in line with the original hypothesis of decrease of functional connectivity of the hippocampal region and provide more specific conjecture about which connections are particularly weakened by this augmented reality (GPS) intervention. Thus, the follow-up studies can narrow down the scope to those detected areas.

## 5. Conclusions

Present study suggests mild decrease of hippocampal functional coupling mainly with temporal areas and cerebellum after three-month intervention with GPS-guided navigation (in contrast to mildly increased functional connectivity observed in control group with no specific intervention). These preliminary findings support the hypothesis of possible harmful effect of long-term usage of GPS technology on our brain functioning when navigation process is externalized to some technical device (e.g., AR glasses). Even if some observed FC changes in experimental group are correlated with duration of the active usage of the GPS device, the expected behavioral effect could not be identified. It should be also noted that some limitations listed above might have affected presented findings that should be therefore interpreted cautiously. Further studies are needed to verify our preliminary findings and establish psychological and neurophysiological consequences of favorite technological aids.

## Figures and Tables

**Table 1:** Functional connectivity of HPC in the baseline TEST session 1 (p-FDR, p < 0.0001). The baseline (before intervention) functional connectivity of the left (L) and right (R) hippocampus in all recruited participants. The ten strongest connections (p-FDR corr. p < 0.0001) are displayed (for complete report of all significant results of FDR corrected at p < 0.05 see Supplementary Table 1).

L Hippocampus			R Hippocampus		
Seed	Analysed_Unit	Statistic	Seed	Analysed_Unit	Statistic
HPC_left	pPaHC_l	T(32)=11.51	HPC_right	pPaHC_r	T(32)=12.55
	Hippocampus_r	T(32)=11.37		Hippocampus_l	T(32)=11.37
	Ver45	T(32)=10.22		LG_l	T(32)=10.35
	LG_l	T(32)=9.64		TOFusC_l	T(32)=10.28
	Cereb45_l	T(32)=9.26		LG_r	T(32)=10.10
	Ver3	T(32)=8.62		Ver3	T(32)=10.06
	LG_r	T(32)=8.49		Amygdala_r	T(32)=9.93
	pPaHC_r	T(32)=8.25		Ver45	T(32)=9.87
	pTFusC_l	T(32)=8.16		Cereb45_l	T(32)=9.63
	Brain-Stem	T(32)=7.94		pPaHC_l	T(32)=9.46

pPaHC_l/r (parahippocampal gyrus, posterior division, left/right); LG_l/r (lingual gyrus, left/right); TOFusC_l (temporal occipital fusiform cortex, left); Ver3 (vermis 3); Ver45 (vermis 4-5); Cereb45_l (cerebellum 4-5, left); pTFusC_l (temporal fusiform cortex, posterior division, left).

**Table 2:** Hippocampal intervention related FC changes (p<0.05, uncorr.). AR glasses intervention related functional connectivity changes of the right (R) and left (L) hippocampi (p<0.05, uncorr.) tested by random-effect second-level analysis.

L Hippocampus					R Hippocampus				
*Seed*	Analysed_Unit	Statistic	p-unc	Cohen's d	*Seed*	Analysed_Unit	Statistic	p-unc	Cohen's d
*HPC_left*	Cereb3_r	T(31)=-3.18	0.003	1.142	*HPC_right*	pTFusC_l	T(31)=-3.49	0.002	1.254
	Brain-Stem	T(31)=-2.71	0.011	0.973		Cereb3_r	T(31)=-3.31	0.002	1.189
	Ver45	T(31)=-2.68	0.012	0.963		pPaHC_l	T(31)=-3.18	0.003	1.142
	Cereb3_l	T(31)=-2.64	0.013	0.948		aPaHC_l	T(31)=-2.69	0.011	0.966
	pPaHC_r	T(31)=-2.53	0.017	0.909		Cereb3_l	T(31)=-2.62	0.013	0.941
	pITG_l	T(31)=-2.35	0.025	0.844		Hippocampus_l	T(31)=-2.08	0.046	0.747
	Amygdala_l	T(31)=-2.15	0.039	0.772					
	Cereb45_r	T(31)=-2.15	0.039	0.772					
	Hippocampus_r	T(31)=-2.08	0.046	0.747					

pTFusC_l (temporal fusiform cortex, posterior division, left); Cereb3_r/l (cerebelum 3, right/left); Cereb45_r (cerebellum 4 and 5, right); pPaHC_r/ l (parahippocampal gyrus, posterior division, right/left); aPaHC_l (parahippocampal gyrus, anterior division, left); Ver45 (vermis 4 and 5); pITG_l (inferior temporal gyrus, posterior division, left).

## Supplementary Materials

Supplementary Materials This includes Supplementary Tables 1-3. Supplementary Table 1 represents a complete list of all significant left/right hippocampal FCs identified in the baseline TEST session and is supplementary to Table 1 (see Results: Resting State fMRI/Baseline Hippocampal FC). Supplementary Table 2 demonstrates the simple intervention effects in functional connections of the right hippocampus calculated in individual groups (FC change between baseline TEST session and RETEST session presented separately for experimental and control group). Please note that the simple effects are calculated only for hippocampal connections in which we previously identified interaction effects between group and intervention (in the previous step of the analysis, see Table 2 of the MS). The table includes also descriptive statistics for each session and group and effect sizes of the presented t-tests. Analogically, Supplementary Table 3 demonstrates the simple intervention effects in functional connections for the left hippocampus.
