# Supplementary material for: Could Prolonged Usage of GPS Navigation Implemented in Augmented Reality Smart Glasses Affect Hippocampal Functional Connectivity?
Source: Biomed Res Int. 2018 Jun 13;2018:2716134. doi: 10.1155/2018/2716134 (PMC6020662; doi:10.1155/2018/2716134)
Supplement: Supplementary Materials — This includes Supplementary Tables 1-3. Supplementary Table 1 represents a complete list of all significant left/right hippocampal FCs identified in the baseline TEST session and is supplementary to Table 1 (see Results: Resting State fMRI/Baseline Hippocampal FC). Supplementary Table 2 demonstrates the simple intervention effects in functional connections of the right hippocampus calculated in individual groups (FC change between baseline TEST session and RETEST session presented separately for experimental and control group). Please note that the simple effects are calculated only for hippocampal connections in which we previously identified interaction effects between group and intervention (in the previous step of the analysis, see Table 2 of the MS). The table includes also descriptive statistics for each session and group and effect sizes of the presented t-tests. Analogically, Supplementary Table 3 demonstrates the simple intervention effects in functional connections for the left hippocampus. [file 2716134.f1.pdf]

Supplementary materials

Supplementary Table 1. Complete results of the FC of the right/ left hippocampus (p<0.05, FDR corrected) measured in all recruited participants in the baseline condition (before intervention).

Legend: *l/L (Left hemisphere), r/R (Right hemisphere); pPaHC (Parahippocampal Gyrus, posterior division); Ver45 (Vermis 4 5); LG (Lingual Gyrus); Cereb45 (Cerebelum 4 5); Ver3 (Vermis 3); pTFusC (Temporal Fusiform Cortex, posterior division); TOFusC (Temporal Occipital Fusiform Cortex); Cereb3 (Cerebelum 3); Precuneous (Precuneous Cortex); ICC (Intracalcarine Cortex); SCC (Supracalcarine Cortex); Ver12 (Vermis 1 2); PC (Cingulate Gyrus, posterior division); aPaHC (Parahippocampal Gyrus, anterior division); toITG (Inferior Temporal Gyrus, temporooccipital part); Ver10 (Vermis 10); pITG (Inferior Temporal Gyrus, posterior division); Cereb6 (Cerebelum 6); Cuneal (Cuneal Cortex); pMTG (Middle Temporal Gyrus, posterior division); MidFG (Middle Frontal Gyrus); Ver6 (Vermis 6); PreCG (Precentral Gyrus); IFGoper (Inferior Frontal Gyrus, pars opercularis); pSMG (Supramarginal Gyrus, posterior division); HG (Heschl's Gyrus); aSMG (Supramarginal Gyrus, anterior division); FO (Frontal Operculum Cortex); PP (Planum Polare); IC (Insular Cortex); IFGtri (Inferior Frontal Gyrus, pars triangularis); CO (Central Opercular Cortex); AG (Angular Gyrus); toMTG (Middle Temporal Gyrus, temporooccipital part)*

**Supplementary Table 1. Functional connectivity of HPC in the baseline TEST session 1 - complete list (p-FDR, p < 0.05)**									
**L Hippocampus**					**R Hippocampus**				
**Seed**	**Analysed_ Unit**	**Statistic**	**p-unc**	**p-FDR**	**Seed**	**Analysed_ Unit**	**Statistic**	**p-unc**	**p-FDR**
HPC_ left	pPaHC_l	T(32)=11.51	0.000	0.000	HPC_ right	pPaHC_r	T(32)=12.55	0.000	0.000
	Hippocampus_r	T(32)=11.37	0.000	0.000		Hippocampus_l	T(32)=11.37	0.000	0.000
	Ver45	T(32)=10.22	0.000	0.000		LG_l	T(32)=10.35	0.000	0.000
	LG_l	T(32)=9.64	0.000	0.000		TOFusC_l	T(32)=10.28	0.000	0.000
	Cereb45_l	T(32)=9.26	0.000	0.000		LG_r	T(32)=10.10	0.000	0.000
	Ver3	T(32)=8.62	0.000	0.000		Ver3	T(32)=10.06	0.000	0.000
	LG_r	T(32)=8.49	0.000	0.000		Amygdala_r	T(32)=9.93	0.000	0.000
	pPaHC_r	T(32)=8.25	0.000	0.000		Ver45	T(32)=9.87	0.000	0.000
	pTFusC_l	T(32)=8.16	0.000	0.000		Cereb45_l	T(32)=9.63	0.000	0.000

	Brain-Stem	T(32)=7.94	0.000	0.000		pPaHC_l	T(32)=9.46	0.000	0.000
	TOFusC_l	T(32)=7.81	0.000	0.000		Cereb45_r	T(32)=9.28	0.000	0.000
	Cereb45_r	T(32)=7.71	0.000	0.000		Precuneous	T(32)=8.73	0.000	0.000
	Thalamus_r	T(32)=7.33	0.000	0.000		Brain-Stem	T(32)=7.89	0.000	0.000
	Cereb3_l	T(32)=7.23	0.000	0.000		Cereb3_r	T(32)=7.37	0.000	0.000
	Precuneous	T(32)=7.07	0.000	0.000		pTFusC_r	T(32)=7.33	0.000	0.000
	Cereb3_r	T(32)=7.01	0.000	0.000		Thalamus_r	T(32)=7.24	0.000	0.000
	Amygdala_l	T(32)=6.95	0.000	0.000		Cereb3_l	T(32)=6.92	0.000	0.000
	Thalamus_l	T(32)=6.73	0.000	0.000		Ver12	T(32)=6.64	0.000	0.000
	ICC_l	T(32)=6.28	0.000	0.000		ICC_l	T(32)=6.43	0.000	0.000
	SCC_l	T(32)=6.03	0.000	0.000		PC	T(32)=6.19	0.000	0.000
	TOFusC_r	T(32)=6.03	0.000	0.000		SCC_l	T(32)=6.05	0.000	0.000
	pTFusC_r	T(32)=5.93	0.000	0.000		pTFusC_l	T(32)=5.76	0.000	0.000
	Amygdala_r	T(32)=5.93	0.000	0.000		ICC_r	T(32)=5.71	0.000	0.000
	ICC_r	T(32)=5.85	0.000	0.000		SCC_r	T(32)=5.67	0.000	0.000
	Ver12	T(32)=5.75	0.000	0.000		Pallidum_r	T(32)=5.44	0.000	0.000
	PC	T(32)=5.70	0.000	0.000		toITG_l	T(32)=5.27	0.000	0.000
	Pallidum_l	T(32)=5.47	0.000	0.000		IFG_oper_r	T(32)=5.14	0.000	0.000
	SCC_r	T(32)=4.72	0.000	0.000		Cereb6_l	T(32)=5.09	0.000	0.000
	aPaHC_l	T(32)=4.68	0.000	0.000		TOFusC_r	T(32)=5.05	0.000	0.000

	Putamen_l	T(32)=4.00	0.000	0.002		pITG_l	T(32)=4.95	0.000	0.000
	toITG_l	T(32)=3.99	0.000	0.002		Thalamus_l	T(32)=4.62	0.000	0.000
	Ver10	T(32)=3.91	0.001	0.002		Putamen_r	T(32)=4.28	0.000	0.001
	pITG_l	T(32)=3.72	0.001	0.003		IC_r	T(32)=4.28	0.000	0.001
	Cereb6_l	T(32)=3.72	0.001	0.003		Amygdala_l	T(32)=4.25	0.000	0.001
	Cuneal_l	T(32)=3.59	0.001	0.004		pMTG_l	T(32)=4.10	0.000	0.001
	pMTG_r	T(32)=3.53	0.001	0.005		IFG_oper_l	T(32)=3.92	0.000	0.002
	MidFG_l	T(32)=3.46	0.002	0.006		pSMG_r	T(32)=-3.82	0.001	0.002
	Ver6	T(32)=3.38	0.002	0.007		aPaHC_r	T(32)=3.78	0.001	0.002
	PreCG_r	T(32)=3.32	0.002	0.008		IFG_tri_r	T(32)=3.78	0.001	0.002
	IFG_oper_l	T(32)=3.26	0.003	0.009		Cuneal_r	T(32)=3.70	0.001	0.003
	pSMG_r	T(32)=-3.13	0.004	0.012		Pallidum_l	T(32)=3.70	0.001	0.003
	Pallidum_r	T(32)=3.11	0.004	0.012		PreCG_r	T(32)=3.66	0.001	0.003
	HG_l	T(32)=3.02	0.005	0.015		FO_l	T(32)=3.65	0.001	0.003
	aSMG_r	T(32)=-2.96	0.006	0.018		dmn.LLP	T(32)=-3.49	0.001	0.004
	FO_l	T(32)=2.92	0.006	0.019		aITG_l	T(32)=3.36	0.002	0.006
	IFG_oper_r	T(32)=2.92	0.006	0.019		MidFG_r	T(32)=3.30	0.002	0.007
	HG_r	T(32)=2.91	0.007	0.019		PP_r	T(32)=3.29	0.002	0.007
	aSMG_l	T(32)=-2.87	0.007	0.020		pSTG_l	T(32)=3.28	0.003	0.007
	Cuneal_r	T(32)=2.82	0.008	0.023		Ver10	T(32)=3.15	0.004	0.010

	PP_l	T(32)=2.74	0.010	0.027		pSMG_l	T(32)=-3.10	0.004	0.011
	IC_l	T(32)=2.70	0.011	0.029		aSMG_r	T(32)=-3.04	0.005	0.013
	IFG_tri_l	T(32)=2.68	0.012	0.030		IFG_tri_l	T(32)=3.00	0.005	0.013
	CO_r	T(32)=2.65	0.012	0.031		CO_r	T(32)=2.93	0.006	0.016
	AG_r	T(32)=-2.64	0.013	0.032		FO_r	T(32)=2.92	0.006	0.016
	IC_r	T(32)=2.61	0.014	0.034		aPaHC_l	T(32)=2.84	0.008	0.019
	PreCG_l	T(32)=2.57	0.015	0.036		FOrb_l	T(32)=2.74	0.010	0.024
	pSMG_l	T(32)=-2.55	0.016	0.037		FOrb_r	T(32)=2.73	0.010	0.024
	CO_l	T(32)=2.53	0.017	0.039		OFusG_l	T(32)=2.72	0.011	0.025
	MidFG_r	T(32)=2.50	0.018	0.041		Cuneal_l	T(32)=2.65	0.013	0.028
	toMTG_r	T(32)=2.42	0.021	0.048		CO_l	T(32)=2.64	0.013	0.028
						Ver6	T(32)=2.64	0.013	0.028
						HG_r	T(32)=2.63	0.013	0.028
						aSMG_r	T(32)=-2.48	0.019	0.040

Supplementary Table 2. Intervention related functional connectivity changes of the right (R) hippocampus (T-test for dependent samples, p<0.05, uncorr.) reported separately for Experimental (Exp.) and Control group. Results for functional connections with significant difference between experimental and control intervention (see Table 2) are reported.

Legend: pTFusC_l (Temporal Fusiform Cortex, posterior division Left); Cereb3_r/l (Cerebelum 3 Right/Left); pPaH_l (Parahippocampal Gyrus, posterior division Left); aPaHC_l (Parahippocampal Gyrus, anterior division Left).

**Supplementary Table 2. Intervention related FC changes - seed: R Hippocampus (p<0.05, uncorr.).**										
**Analysed_ Unit**	**Exp. Test Mean (SD)**	**Exp. Retest Mean (SD)**	**T-test (df=16)**	**p value**	**Cohen's d**	**Control Test Mean (SD)**	**Control Retest Mean (SD)**	**T-test (df=15)**	**p value**	**Cohen's d**
pTFusC_l	0.445 (0.302)	0.232 (0.265)	3.21	0.006	0.803	0.307 (0.434)	0.448 (0.318)	-1.74	0.103	0.449
Cereb3_r	0.688 (0.283)	0.516 (0.365)	2.45	0.026	0.613	0.320 (0.413)	0.500 (0.379)	-2.14	0.049	0.553
pPaHC_l	0.656 (0.255)	0.516 (0.273)	2.40	0.029	0.600	0.441 (0.366)	0.568 (0.306)	-1.96	0.069	0.506
aPaHC_l	0.365 (0.462)	0.063 (0.305)	2.68	0.016	0.670	0.076 (0.315)	0.207 (0.277)	-1.68	0.114	0.434
Cereb3_l	0.440 (0.339)	0.234 (0.292)	2.86	0.011	0.715	0.313 (0.268)	0.350 (0.334)	-0.57	0.575	0.147
Hippocampus_ l	0.740 (0.217)	0.659 (0.241)	1.19	0.250	0.298	0.499 (0.369)	0.675 (0.295)	-1.61	0.127	0.416

Supplementary Table 3. Intervention related functional connectivity changes of the left (L) hippocampus (T-test for dependent samples, p<0.05, uncorr.) reported separately for Experimental (Exp.) and Control group. Results for functional connections with significant difference between experimental and control intervention (see Table 2) are reported.

Legend: Cereb3_r/l (Cerebelum 3 Right/Left); Cereb45_r (Cerebelum 4 and 5 Right); pPaH_r (Parahippocampal Gyrus, posterior division Right); Ver45 (Vermis 4 and 5); pITG l (Inferior Temporal Gyrus, posterior division Left)

Supplementary Table 3. Intervention related FC changes - seed: L Hippocampus (p<0.05, uncorr.).

Analysed_ Unit	Exp. Test Mean (SD)	Exp. Retest Mean (SD)	T-test (df=16)	p value	Cohen's d	Control Test Mean (SD)	Control Retest Mean (SD)	T-test (df=15)	p value	Cohen's d
Cereb3_r	0.640 (0.342)	0.519 (0.401)	1.55	0.141	0.388	0.365 (0.444)	0.571 (0.316)	-2.95	0.010	0.762
Brain-Stem	0.565 (0.273)	0.394 (0.217)	2.37	0.031	0.593	0.322 (0.325)	0.496 (0.327)	-1.64	0.122	0.423
Ver45	0.683 (0.249)	0.593 (0.327)	1.78	0.094	0.445	0.502 (0.392)	0.649 (0.348)	-1.75	0.101	0.452
Cereb3_l	0.538 (0.338)	0.370 (0.378)	1.79	0.092	0.448	0.412 (0.338)	0.573 (0.378)	-2.09	0.054	0.540
pPaHC_r	0.710 (0.331)	0.608 (0.345)	1.23	0.236	0.308	0.468 (0.470)	0.684 (0.298)	-2.32	0.035	0.599
pITG_l	0.014 (1.088)	0.147 (0.305)	-0.526	0.606	0.132	0.065 (0.250)	0.189 (0.220)	-1.76	0.100	0.454
Amygdala_l	0.490 (0.292)	0.428 (0.195)	0.73	0.477	0.183	0.368 (0.418)	0.551 (0.364)	-2.20	0.044	0.568
Cereb45_r	0.607 (0.341)	0.502 (0.362)	1.45	0.167	0.363	0.423 (0.401)	0.559 (0.311)	-1.45	0.168	0.374
Hippocampus_r	0.740 (0.217)	0.659 (0.241)	1.19	0.250	0.298	0.499 (0.369)	0.675 (0.295)	-1.61	0.127	0.416
